# Supplementary material for: Protection of K18-hACE2 Mice against SARS-CoV-2 Challenge by a Capsid Virus-like Particle-Based Vaccine
Source: Vaccines (Basel). 2024 Jul 12;12(7):766. doi: 10.3390/vaccines12070766 (PMC11281552; doi:10.3390/vaccines12070766)
Supplement: Supplementary file 1 [file vaccines-12-00766-s001.zip › vaccines-3041889-supplementary.pdf]

Myeni *et al.* **I Supplementary Figure S1**

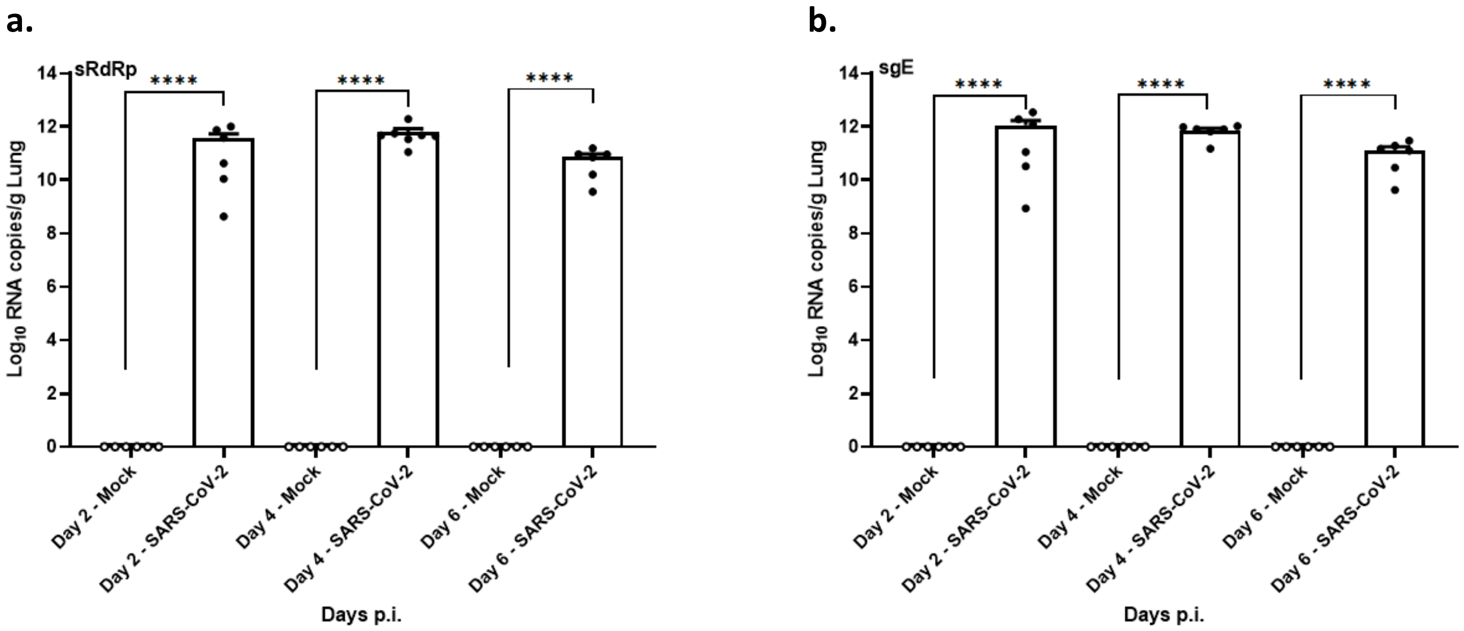

Figure S1. **SARS-CoV-2/Leiden-008 viral loads in the lung after infection in K18-hACE2 mice.** **a**. Viral sub-genomic RNA and **b.** genomic RNA in the lungs at day 2, 4 and 6 post infection. Viral RNA levels were determined by RT-qPCR using primers targeting the E (subgenomic RNA) and RdRp (genomic RNA) coding regions. PGK1 and lung weights were used to normalize the RNA copy levels. The mean per group (n = 6) and the virus RNA copies per gram of lung tissue are presented with symbols for each mouse and the error bars represent standard error of mean. ns: not significant, *p<0.05, ** p<0.01, *** p<0.001, **** p<0.0001.

Myeni *et al.* **I Supplementary Figure S2**

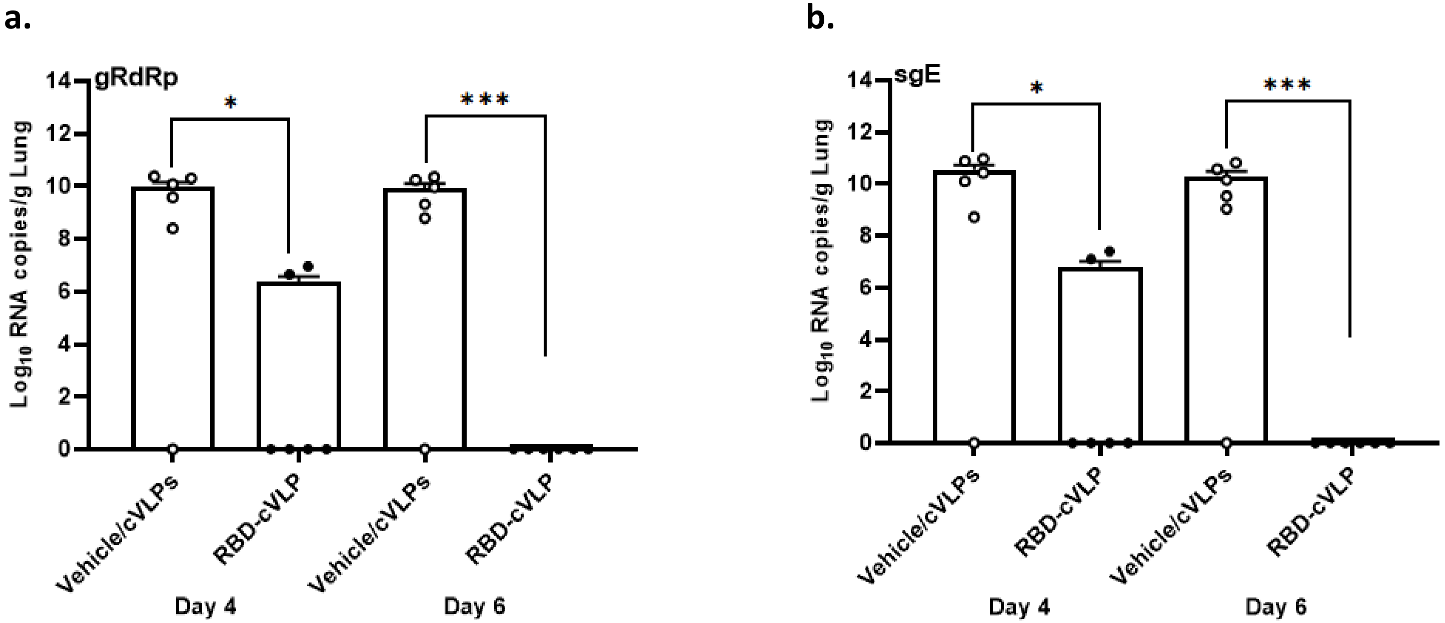

Figure S2. **Viral loads in the lung after challenge**. **a**. Viral sub-genomic RNA and **b.** genomic RNA in the lungs of SARS-CoV-2 challenged mock (Vehicle/cVLPs) or RBD-cVLP vaccinated K18-hACE2 mice on days 4 and 6 post-challenge. Viral RNA levels were determined by RT-qPCR using primers targeting the E (subgenomic RNA) and RdRp (genomic RNA) coding regions. PGK1 and lung weights were used to normalize the RNA copy levels. The mean per group (n = 6) and the virus RNA copies per gram of lung tissue are presented with symbols for each mouse and the error bars represent standard error of mean. ns: not significant, *p<0.05, ** p<0.01, *** p<0.001, **** p<0.0001.

Myeni *et al.* **I Supplementary Table S1**

Vaccination type	Extent	Alveolar interstitial inflammation			Perivascular mixed inflammatory cell infiltrate and edema	Necrosis	Intra-alveolar neutrophils	Intra-alveolar macrophages	Hyaline membranes per alveolus	Intra-alveolar fluid	Intra-alveolar hemorrhage	Alveolar septal thickening	Combined path score
		Neuts	Macs	Lymphocytes									
**AV14**	**1,80**	**0,20**	**2,00**	**1,00**	**0,20**	**0,00**	**0,00**	**1,00**	**0,00**	**0,00**	**0,00**	**1,80**	**12,80±8,52**
Animal 6	2	1	3	1	0	0	0	1	0	0	0	3	**18**
Animal 7	2	0	3	2	1	0	0	1	0	0	0	3	**20**
Animal 8	3	0	3	1	0	0	0	1	0	0	0	2	**21**
Animal 9	1	0	0	0	0	0	0	1	0	0	0	0	**1**
Animal 10	1	0	1	1	0	0	0	1	0	0	0	1	**4**
**Vehicle/CLP**	**2,00**	**1,00**	**2,80**	**1,20**	**2,20**	**0,00**	**1,20**	**2,20**	**0,40**	**0,20**	**0,00**	**2,80**	**29,00±13,08**
Animal 11	2	1	3	1	3	0	2	3	1	0	0	3	**34**
Animal 12	2	1	3	2	3	0	1	3	0	1	0	3	**34**
Animal 13	2	1	3	1	1	0	0	1	0	0	0	2	**18**
Animal 14	1	1	2	0	2	0	1	2	0	0	0	3	**11**
Animal 15	3	1	3	2	2	0	2	2	1	0	0	3	**48**

Combined path score is indicated as the mean value from 5 different animals ± standard deviation; Semi-quantitative score parameters: Extent: 0=none/minimal (<5%), 1=focal (5-33%), 2=multifocal (33-66%), 3=diffuse (66-100%); Alveolar interstitial inflammation, perivascular mixed inflammatory cell infiltrates and edema, necrosis, intra-alveolar neutrophils, macrophages, and hemorrhage: 0=none, 1=mild, 2=moderate, 3=severe; Alveolar septal thickening: 0=none, 1=2-fold increased, 2=2-4-fold increased, 3=more than 4-fold increased compared with unaffected septa; Hyaline membranes, intra-alveolar proteinaceous fluid: 0=none, 1=1, 2=more than 1 per alveolus.
Abbreviations: Neuts=neutrophils; Macs=macrophages; path=pathology

Table S1. **Lung histopathology scores from RBD-cVLP (AV14) and mock/Vehicle/CLP (Vehicle/cVLPs)- immunized K18-hACE2 mice on day 4 post infection with SARS-CoV-2.** Listed are all semi-quantitatively scored lung lesions per animal and the calculated combined lung pathology scores. Group means (all scores) and standard error of the mean (combined score only) are indicated in bold.

Vaccination type	Extent	Alveolar interstitial inflammation			Perivascular mixed inflammatory cell infiltrate and edema	Necrosis	Intra-alveolar neutrophils	Intra-alveolar macrophages	Intra-alveolar lymphocytes	Hyaline membranes per alveolus	Intra-alveolar fluid	Intra-alveolar hemorrhage	Alveolar septal thickening	Combined path score
		Neuts	Macs	Lymphocytes										
**AV14**	**2,4**	**1,4**	**1,4**	**0,6**	**0,0**	**0,0**	**0,0**	**0,0**	**0,0**	**0,8**	**0,0**	**0,0**	**1,8**	**16,0±7,97**
Animal 6	1	0	1	0	0	0	0	0	0	0	0	0	0	**1**
Animal 7	3	1	2	0	0	0	0	0	0	1	0	0	2	**18**
Animal 8	3	2	1	1	0	0	0	0	0	1	0	0	2	**21**
Animal 9	3	2	1	1	0	0	0	0	0	1	0	0	3	**24**
Animal 10	2	2	2	1	0	0	0	0	0	1	0	0	2	**16**
**Vehicle/CLP**	**3,0**	**2,0**	**2,5**	**1,0**	**2,8**	**0,2**	**2,0**	**1,8**	**1,3**	**1,8**	**1,8**	**0,2**	**2,8**	**61,8±5,56**
Animal 11	3	2	3	1	3	0	2	2	1	2	2	0	3	**63**
Animal 12	3	2	2	1	3	0	2	1	2	2	2	0	2	**57**
Animal 13	3	2	3	1	3	0	2	2	2	2	2	0	3	**66**
Animal 14	3	2	2	1	3	0	2	2	1	1	1	0	3	**54**
Animal 15	3	2	2	1	3	1	2	2	1	2	3	1	3	**69**

Combined path score is indicated as the mean value from 5 different animals ± standard deviation; Semi-quantitative score parameters: Extent: 0=none/minimal (<5%), 1=focal (5-33%), 2=multifocal (33-66%), 3=diffuse (66-100%); Alveolar interstitial inflammation, perivascular mixed inflammatory cell infiltrates and edema, necrosis, intra-alveolar neutrophils, macrophages, and hemorrhage: 0=none, 1=mild, 2=moderate, 3=severe; Alveolar septal thickening: 0=none, 1=2-fold increased, 2=2-4-fold increased, 3=more than 4-fold increased compared with unaffected septa; Hyaline membranes, intra-alveolar proteinaceous fluid: 0=none, 1=1, 2=more than 1 per alveolus.
Abbreviations: Neuts=neutrophils; Macs=macrophages; path=pathology

Table S2. Lung histopathology scores from RBD-cVLP (AV14) **and** mock/Vehicle/CLP (Vehicle/cVLPs) **- K18-hACE2 mice on day 6 post infection with SARS-CoV-2.** Listed are all semi-quantitatively scored lung lesions per animal and the calculated combined lung pathology scores. Group means (all scores) and standard error of the mean (combined score only) are indicated in bold.
